# When cells get in the flow

**DOI:** 10.7554/eLife.77309

**Published:** 2022-02-28

**Authors:** Giovanna M Collu, Marek Mlodzik

**Affiliations:** 1 Department of Cell, Developmental and Regenerative Biology, Graduate School of Biomedical Sciences, Icahn School of Medicine at Mount Sinai New York United States

**Keywords:** eye development, mechanics, pattern formation, retinal development, tissue organization, *D. melanogaster*

## Abstract

New imaging approaches question a long-standing model for how the eyes of fruit flies acquire their geometric patterning.

**Related research article** Gallagher KD, Mani M, Carthew RW. 2022. Emergence of a geometric pattern of cell fates from tissue-scale mechanics in the *Drosophila* eye. *eLife* **11**:e72806. doi: 10.7554/eLife.72806.

Take a close look at the skin of a bird or a mammal, and you will observe an impressively regular pattern of hair follicles or feathers interspersed with epidermal skin cells (Ho et al., 2019; Sick et al., 2006; Stark et al., 2007). A strikingly similar organization exists in the eyes of the developing fruit fly *Drosophila*, with clusters of photoreceptor neurons regularly distributed amongst epithelial support cells. Each cluster will go on to form one facet in the compound adult eye (Heberlein and Treisman, 2000).

Several theoretical models have been proposed to explain how such intricate spacing patterns emerge. The ‘reaction-diffusion model’ suggested by Alan Turing, for example, postulates that cells developing as a feather bud secrete an inhibitor around themselves to stop equivalent structures from forming too closely (Turing, 1997; Figure 1A). Now, in eLife, Kevin Gallagher, Madhav Mani and Richard Carthew – who are all based at Northwestern University – report on new observations that challenge this view, proposing an alternative mechanism that relies on complex cell movements during development (Gallagher et al., 2022).

**Figure 1.**
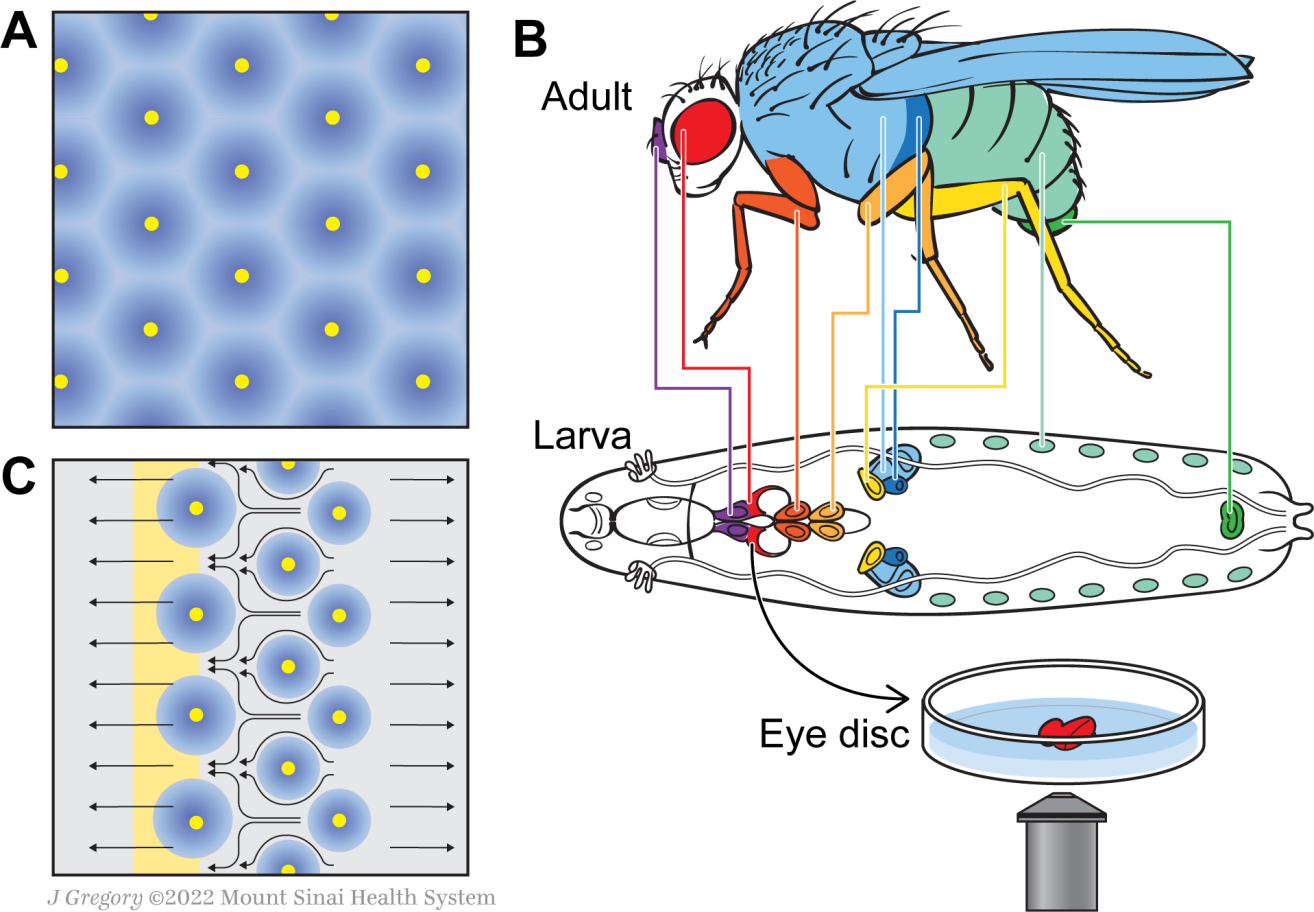
A new model for how the fly eye acquires its patterning during development. (**A**) In birds and mammals, the epidermis features a geometric patterning, with feathers or hair follicles having regular spacing between them. According to the reaction-diffusion model, this organisation emerges because of short-range activators (yellow) and long-range inhibitors (blue) limiting the sites at which feathers or hair follicles form. (**B**) During the larval stage, the cells that will form structures in the adult fly develop in sacs of cells called imaginal discs, which can be identified by their shape and position in the larva. Colour-coding shows the correspondence between individual discs and adult structures (e.g. the red imaginal discs in the larvae will mature into eyes – also in red). Disc dissection, culture, and direct imaging allow visualization of developmental patterning processes, as shown by Gallagher and colleagues for the eye disc. (**C**) Gallagher et al. propose an updated model to explain the geometric patterning of the eye disc. An activator (atonal, yellow), and Scabrous (a possible secreted inhibitor, blue) are expressed by early photoreceptor clusters. The yellow stripe on the left represents the morphogenetic furrow moving from the posterior (right) to the anterior side of the disc (left). Cells to the left of the furrow constrict and those to the right expand, driving cell flow in the pattern shown by the black arrows. This cellular movement ensures regularly spaced photoreceptor clusters.

During the fruit fly’s larval stages, the cells that will form adult structures develop in isolated sacs called imaginal discs. During metamorphosis, the larval tissue is degraded and the discs expand and fuse to create adult structures (Figure 1B). This process is difficult to visualize as it happens inside the developing insect. Traditional approaches involve dissecting and fixing the tissue, which can only provide ‘snapshots’ at specific time points. As a response, Gallagher et al. set up a new system that allowed them to image cellular behavior ‘live’ in developing larvae, achieving high spatial and temporal resolution. To do this, they isolated discs from genetically modified flies that express fluorescent cell adhesion proteins, and cultured these structures ex vivo; this allowed the team to track cell outlines in developing larvae, and to follow their movement over time.

Gallagher et al. applied their technique to the eye disc, whose development is controlled by a wave of signals – the morphogenetic furrow – spreading over the disc from one end to the other. The cells constrict as the furrow passes over them, but it was assumed that they remained stationary through this process. Specific cell fates, and therefore the final patterning of the eye, were thought to emerge from the signaling molecules and events associated with the furrow (Heberlein and Treisman, 2000).

Yet, the results from Gallagher et al. contradict that long-standing assumption, showing instead that cells in the eye disc are very dynamic. As individual cells rearrange themselves with respect to their neighbors, they move away from any potential diffusing signal they may have been exposed to initially; this means that the Turing’s reaction-diffusion model cannot sufficiently explain how the final geometric pattern emerges in the eye. Instead, these individual motions create an overall, strikingly cohesive ‘flow’, which pushes cells towards the morphogenetic furrow.

Gallagher et al. then analyzed the cell flow mathematically and calculated how quickly cells moved depending on their position relative to the furrow. This revealed that cells undergo periodic phases of fast and slow movement, with these oscillations correlating with the precise spacing of photoreceptor clusters. Cells in defined areas behind and in front of the furrow respectively dilate or constrict, generating forces that create the periodic cell flow: the dilation pushes the cells to move away while the constriction serves as a ‘sink’ that draws the cells towards it. This means that cells flow into the spaces between photoreceptor clusters, ensuring that these are regularly spaced out (Figure 1C).

Next, Gallagher et al. examined the role of Scabrous, a protein secreted during development that affects the geometric spacing of photoreceptor clusters in unknown ways (Baker et al., 1990; Mlodzik et al., 1990). In mutant tissue lacking Scabrous, cell dilation decreases; cell flow becomes disrupted, leading to irregular spacing and tissue organization. How Scabrous regulates the dilation and flow of cells remains unclear, but the observation confirms how important organized cell flow is for tissue patterning to emerge.

As cell flow becomes one of the mechanisms known to regulate precise tissue organization, many exciting questions emerge: how is this process controlled genetically? Do signaling molecules from the morphogenetic furrow regulate cell dilation and constriction, as well as cell identity and inhibitor production? Or does cell flow regulate gene expression directly within the furrow? The new imaging approach by Gallagher et al. will help to address these questions, allowing researchers to track how signaling and cell flow cooperate to organize the eye tissue pattern.

A few recent studies focusing on the later stages of eye development in *Drosophila*, as well as other tissues, also highlight that cells need to move together and relative to each other (known as tissue fluidity) for an organism to develop (Founounou et al., 2021; Aigouy et al., 2010; Mongera et al., 2018). These findings are ushering in a new phase of research in organogenesis and developmental biology, allowing alternative models to emerge based on tissue fluidity, cell flow, and tissue organization.

## References

[bib1] Aigouy B, Farhadifar R, Staple DB, Sagner A, Röper J-C, Jülicher F, Eaton S (2010). Cell flow reorients the axis of planar polarity in the wing epithelium of *Drosophila*. Cell.

[bib2] Baker NE, Mlodzik M, Rubin GM (1990). Spacing differentiation in the developing *Drosophila* eye: a fibrinogen-related lateral inhibitor encoded by scabrous. Science.

[bib3] Founounou N, Farhadifar R, Collu GM, Weber U, Shelley MJ, Mlodzik M (2021). Tissue fluidity mediated by adherens junction dynamics promotes planar cell polarity-driven ommatidial rotation. Nature Communications.

[bib4] Gallagher KD, Mani M, Carthew RW (2022). Emergence of a geometric pattern of cell fates from tissue-scale mechanics in the *Drosophila* eye. eLife.

[bib5] Heberlein U, Treisman JE, Elizabeth Fini M (2000). Vertebrate Eye Development.

[bib6] Ho WKW, Freem L, Zhao D, Painter KJ, Woolley TE, Gaffney EA, McGrew MJ, Tzika A, Milinkovitch MC, Schneider P, Drusko A, Matthäus F, Glover JD, Wells KL, Johansson JA, Davey MG, Sang HM, Clinton M, Headon DJ (2019). Feather arrays are patterned by interacting signalling and cell density waves. PLOS Biology.

[bib7] Mlodzik M, Baker NE, Rubin GM (1990). Isolation and expression of scabrous, a gene regulating neurogenesis in *Drosophila*. Genes & Development.

[bib8] Mongera A, Rowghanian P, Gustafson HJ, Shelton E, Kealhofer DA, Carn EK, Serwane F, Lucio AA, Giammona J, Campàs O (2018). A fluid-to-solid jamming transition underlies vertebrate body axis elongation. Nature.

[bib9] Sick S, Reinker S, Timmer J, Schlake T (2006). WNT and DKK determine hair follicle spacing through a reaction-diffusion mechanism. Science.

[bib10] Stark J, Andl T, Millar SE (2007). Hairy math: insights into hair-follicle spacing and orientation. Cell.

[bib11] Turing MA (1997). The chemical basis of morphogenesis. Philosophical Transactions of the Royal Society of London. Series B, Biological Sciences.

